# Beyond survival: Prioritizing the unmet mental health needs of pregnant and postpartum women and their caregivers

**DOI:** 10.1371/journal.pgph.0002782

**Published:** 2024-02-05

**Authors:** Laura Fitzgerald, Shanon McNab, Pasqueline Njau, Prabha Chandra, Phiona Koyiet, Rebecca Levine, Pandora Hardtman, Suzanne Stalls

**Affiliations:** 1 MOMENTUM Country and Global Leadership, Jhpiego, Washington, District of Columbia, United States of America; 2 Calmind Foundation, Nairobi, Kenya; 3 Department of Psychiatry, the National Institute of Mental Health and Neuro Sciences, Bangalore, India; 4 Global Technical Resource Team: Disaster Management, World Vision International, Nairobi, Kenya; 5 Global Health Practice, Palladium, Washington, District of Columbia, United States of America; 6 Technical Leadership Office, Jhpiego, Baltimore, Maryland, United States of America; Harare Central Hospital, ZIMBABWE

Two alarming and consequential truths were exposed and exacerbated by the COVID-19 pandemic. First, a global mental health crisis endangers the well-being of families, communities, countries, and the personnel that support them. This crisis has particularly damaging implications for adolescents, pregnant and postpartum women, mothers, and health care workers [1]. Second, improvements in the health, survival, and well-being of pregnant, childbearing, and postpartum women around the world have stalled. A recent global report found that the much-celebrated improvements in maternal mortality ratios have, in fact, stagnated or worsened in sub-Saharan and Northern Africa, Oceania (excluding Australia and New Zealand); Western, Eastern, and South-Eastern Asia; Europe and Northern America; and Latin America and the Caribbean between 2016 and 2020 [2]. The evidence clearly shows that the mental health and survival of pregnant and postpartum women have been sidelined.

Women’s health care providers are suffering as well, with anxiety, burnout, and secondary trauma well documented among nurses and midwives predating the COVID-19 pandemic [3, 4]. These statistics have worsened since the pandemic, as health care professionals around the world—including nurses and midwives—became ill, lost their lives, or left the profession, leaving an even greater care burden for those who remained [5] and further compromising their mental health. A recent study in the U.S. found that health care work is more strongly associated with suicide risk among female workers, who make up the majority of both the nursing and midwifery professions [6]. It becomes a vicious cycle; as health care shortages and poor working conditions exact a heavy toll on providers’ mental health, they are less able to meet the physical and psycho-emotional needs of the women and babies in their care, who then experience worse physical and mental health outcomes. Urgent, accelerated, and evidence-based investments in mental health must be prioritized.

The perinatal period is a life phase marked by profound physical, psychological, and interpersonal growth and change, a combination of factors that put pregnant and postpartum women at elevated risk of mental disorders [7, 8]. These changes are unique to the perinatal period; they are not experienced during any other time of life. The transition to motherhood brings unique stressors, often including infant feeding challenges, sleep deprivation, near constant caregiving responsibilities, and healing from the birth experience. Common perinatal mental disorders (CPMDs), such as anxiety, depression, and somatic disorders, are the most frequent complications of pregnancy, childbirth, and the postpartum period [9, 10]. CPMDs affect a woman’s physical health during the perinatal period as well as her long-term mental and physical health, functioning, and quality of life [11]. And, unlike the health consequences of mental health conditions in other populations, when a woman experiences a mental health challenge in the perinatal period, the impact extends beyond herself. A woman’s physical and mental health are inextricably linked to her baby’s well-being. When a woman suffers, her child, or children, suffer too. Perinatal mental health conditions are associated with a number of adverse outcomes in newborns and children.

Given the urgency of the current maternal mortality crisis, and the fact that the treatment gap for people with mental health disorders is as high as a staggering 90% in many low- and middle-income country settings, perinatal mental health should be seen as a core element in improving maternal survival and well-being [12]. Yet, despite mounting evidence of the impact of perinatal mental health on women and children, the integration of prevention and treatment of CPMDs into maternal, newborn, and child health programs is slow and limited. This is particularly true in low- and middle-income countries where data on perinatal mental health, like data on other maternal health outcomes, is sparse and varied, and significant gaps in meaningful public health commitments to mental health remain [13, 14]. And while lay health workers, like community health workers or peer volunteers, in programs such as the World Health Organization’s (WHO) Thinking Healthy can be tremendous assets, the maternal and newborn health community still lacks the detailed implementation learning around what works, where, and why when integrating perinatal mental health interventions into existing health systems at scale [15].

Prevailing gendered norms about the “naturalness” of motherhood and the expectation of uncomplicated joy at the birth of a child compound the silence, stigma, and shame that often accompanies the experience of mental health issues and further prevents the urgent action that is needed at all levels. The perinatal period is a phase of life in which individuals have repeated contact with health care providers. Prioritizing mental health during pregnancy and the postpartum period is essential not just to improve maternal, child, and familial survival and well-being, but because women must be supported to adapt to the significant changes that take place during this period in order to fully assume critical roles in the economy, politics, and social development. Harnessing existing perinatal service delivery platforms to address women’s mental health needs, in addition to their physical health needs, is thus an imperative for individual and societal impact that countries cannot afford to miss. While meeting the pressing physical and mental health needs of all populations, across all service delivery areas, is a mandate for all country governments, maternal and newborn health has consistently been undervalued (as evidenced by the maternal mortality data presented at the beginning of this article).

Midwives, who can meet nearly 90% of the need for essential sexual, reproductive, maternal, newborn, and adolescent health interventions [16], and other obstetric and newborn care providers must be engaged as part of the solution to the crises outlined above. However, community health workers, nurses, midwives, the vast majority of whom are women [17], also face significant societal and professional barriers that hamper their ability to provide high-quality, safe care. Multiple, often deeply gender-based factors make their jobs unacceptably difficult. These factors include dire staffing shortages, insufficient supplies, equipment, and space, discrimination, violence in the workplace, inadequate representation at leadership and policy levels, poor and unequal compensation, minimal professional support and continued educational opportunities, and disease outbreaks, among others [18]. On top of these professional challenges, caregiving and familial expectations and demands at home lead to exhaustion, “compassion fatigue,” attrition, and other mental health concerns [16, 19]. It is a dynamic, deeply systemic and interconnected problem.

Health systems can better support these providers by utilizing their insights and priorities in the development of interventions, strengthening their pre and in-service training on mental health, introducing culturally validated screening tools, and acknowledging and addressing providers’ mental health needs. Health systems can also create policies against harassment or violence in the workplace, conduct gender analyses to ensure pay equity, offer equal opportunities for caregiving leave, and support women-led peer support, mentoring, and leadership development mechanisms [17].

Pregnant, birthing, and postpartum women who are treated with disrespect, or who experience obstetric trauma or perinatal loss, are at increased risk for mental health issues [20]. Birthing women deserve informed, evidence-based, empathetic care to protect their mental health, the kind of care health workers can only provide if they themselves have the requisite resources and interprofessional respect and support to do their jobs to the best of their abilities. It stands to reason that improving the well-being of maternal and newborn care providers—through better, fairer, more equitable working conditions—should also lead to better physical and mental health outcomes for women. We recognize the absence of rigorous data to confirm these causal pathways. However, the fact that these are not robust areas of thoughtful inquiry is important to note. It suggests that medical and public health bodies and institutions have not deemed them significant enough to warrant meaningful exploration. We know from the medical historical record that women have largely been overlooked in published medical and pharmaceutical research, with men serving as the standard point of reference [21].

While some evidence does exist about the severity and consequences of these complex issues, as well as what works to address them, we are now faced with an opportunity to prioritize women’s mental and physical health care by linking these critical issues with more and better data. We need more studies to understand the gaps, challenges, and unaddressed mental health needs that women and providers face. We need more evidence generated from diverse, lower-resource settings that work within maternal and newborn systems, such as in group antenatal or postnatal care, or conducting sessions with women during their pregnancy and postpartum visits. We also need more evidence about CPMDs beyond postnatal depression, about strategies that respond to women’s expressed desires, and about integrated approaches that improve health for both women and children [15]. In other words, we must thoughtfully explore women’s and health workers’ contextualized experiences, preferences, and needs and prioritize them in designing and implementing maternal health programs that respond to their perceptions and expressions of mental health issues. While this may sound like basic formative research, it is still lacking. We can learn from, build upon, and introduce effective low-intensity interventions now. For example, the ultra-short mental health screening tools that are used in some high-volume clinics in South Africa could be assessed for possible adaptation to other contexts [22].

Evidence from high-income countries suggests that advocacy from women with lived experience, and their loved ones, can be a powerful tool for catalyzing investment and scale-up of effective, relatively simple perinatal mental health interventions [23]. But until we acknowledge that the perinatal period poses unique challenges and opportunities—not yet fully understood as distinct from broader mental health programming—women will go largely unsupported.

Investing in enabling environments that make it both possible and desirable to provide high-quality, respectful, person-centered care across the life cycle will not only enhance providers’ contributions, but will also create a more welcoming and safe health system for women and families. Such environments would include effective and supportive supervisory systems, functional referral mechanisms, and available services for women requiring advanced care. Additionally, equipping providers with the basic knowledge and skills in gender transformative care, trauma-informed care, and mental health and counseling can help them to prevent, identify, address, and/or refer mental health issues in the perinatal period. Community assets should be leveraged so the wisdom and experience of community leaders, elders, and peers can offer a wider network of informed, compassionate support that helps to shift the stigmatizing norms and expectations of how a mother should feel or react after birth [24]. We can take the mental health needs of providers seriously by managing their psychological risks; protecting and promoting their mental health through training, skills building, and empowerment; and supporting those with mental health conditions per guidance from the International Labour Organization [25]. Introducing tools such as WHO’s Self-Help Plus (SH+) can help health workers manage stress, reduce burnout, and promote resilience [26]. But health systems also need to find ways of reducing stress, providing mental health leave, and ensuring equitable workplaces that are free of harassment and fear.

The perinatal period provides a unique opportunity to effectively reach women and their families; many women are highly motivated to address their own health and behaviors during pregnancy and are even more likely to attend follow-up visits for their babies than for themselves. The case for investing in the mental health needs of perinatal women is so compelling because of the largely unmet, substantial, and increased need during this period, because making a meaningful impact can be achieved through existing service delivery platforms and patient-provider touchpoints, and because the stakes—in terms of morbidity and mortality—are unconscionably high if we do not act. The vision is clear: whole-person, family-centered, context-responsive maternal health care that recognizes the fact that there is no health without mental health, and health systems that meaningfully address communities’ mental health needs while paying specific attention to the particular mental health challenges of the perinatal period. Realizing this vision will require a powerful transformation of “business as usual”—an overturning of inefficient, gendered, and dysfunctional systems to center the health and well-being of millions of women and families for generations to come. The question now is whether we are ready to truly commit to this transformation.
